# Advances in the Pathophysiology and Management of Cancer Pain: A Scoping Review

**DOI:** 10.3390/cancers18020259

**Published:** 2026-01-14

**Authors:** Giustino Varrassi, Antonella Paladini, Y Van Tran, Van Phong Pham, Ameen A. Al Alwany, Giacomo Farì, Annalisa Caruso, Marco Mercieri, Joseph V. Pergolizzi, Alan D. Kaye, Frank Breve, Alberto Corriero, Christopher Gharibo, Matteo Luigi Giuseppe Leoni

**Affiliations:** 1 Fondazione Paolo Procacci, 00193 Roma, Italy; 2 College of Medicine, University of Baghdad, Baghdad 10071, Iraq; ameen.a@comed.uobaghdad.edu.iq; 3 Department of Medicina Clinica, Sanità Pubblica, Scienze della Vita e dell’Ambiente (MESVA), University of L’Aquila, 67100 L’Aquila, Italy; antonella.paladini@univaq.it; 4 Department of Anesthesiology and Pain Medicine, Binh Dan Hospital, Ho Chi Minh City 70000, Vietnam; pbeiu25003@phd.hcmiu.edu.vn (Y.V.T.); pbeiu25005@phd.hcmiu.edu.vn (V.P.P.); 5 Department of Experimental Medicine (Di.Me.S.), University of Salento, 73100 Lecce, Italy; giacomo.fari@unisalento.it; 6 Department of Surgery, Azienda Socio-Sanitaria Territoriale (ASST) Lodi, 26900 Lodi, Italy; annalisacarusomd@gmail.com; 7 Department of Medical and Surgical Sciences and Translational Medicine, Sapienza University of Rome, 00189 Rome, Italy; marco.mercieri@uniroma1.it (M.M.); matteolg.leoni@gmail.com (M.L.G.L.); 8 NEMA Research Inc., Naples, FL 34108, USA; jpergolizzi@nemaresearch.net; 9 Department of Anesthesiology, LSU Health, Shreveport, LA 71115, USA; alan.kaye@lsuhs.edu; 10 School of Pharmacy, Temple University, Philadelphia, PA 19122, USA; f.breve@comcast.net; 11 Department of Interdisciplinary Medicine, University of Bari Aldo Moro, 70121 Bari, Italy; alberto.corriero@gmail.com; 12 Department of Anesthesiology, NYU Grossman School of Medicine, New York, NY 10016, USA; christopher.gharibo@nyulangone.org

**Keywords:** cancer pain, tumor–neuron interactions, pain management, neuromodulation, artificial intelligence, precision medicine

## Abstract

**Background/Objectives:** Cancer pain affects 55–95% of patients with advanced malignancy, representing a complex syndrome involving nociceptive, neuropathic and nociplastic mechanisms. Despite therapeutic advances, two-thirds of patients with metastatic cancer experience inadequate pain control. This scoping review synthesizes recent advances in cancer pain pathophysiology and management, focusing on molecular and cellular mechanisms, emerging pharmacological, interventional and technological therapies and key evidence gaps to inform future precision-based pain management strategies. **Methods:** Following PRISMA-ScR methodology, we searched PubMed, Embase, Scopus, and Web of Science for studies published between January 2022 and September 2025. After screening 3412 records, 278 studies were included and analyzed across different domains: biological mechanisms, pharmacological management, interventional and neuromodulatory approaches, radiotherapy developments, and digital health innovations. **Results:** Recent mechanistic research reveals cancer pain arises from tumor–neuron–immune crosstalk, with malignant cells secreting neurotrophic factors that promote axonal sprouting and nociceptor sensitization. Genetic polymorphisms and epigenetic modifications contribute to inter-individual pain variability. Management strategies are evolving toward multimodal precision medicine: NSAIDs and opioids remain foundational, complemented by adjuvant agents and interventional procedures including nerve blocks, intrathecal delivery, and neuromodulation (spinal cord and dorsal root ganglion stimulation). Stereotactic body radiotherapy demonstrates superior analgesic durability versus conventional approaches. Digital health innovations, such as mobile applications, remote monitoring, wearables, and AI-enabled predictive models, enable continuous assessment and personalized treatment optimization. **Conclusions:** Cancer pain management is transitioning toward mechanism-based precision medicine integrating biological insights, advanced interventional techniques, and digital technologies. However, implementation challenges persist, including limited randomized trials for interventional approaches, the incomplete external validation of AI tools, and digital health equity concerns. Future research must prioritize prospective controlled studies and equitable integration into routine care.

## Simple Summary

Cancer pain is a debilitating symptom affecting most patients with advanced cancer, yet many continue to suffer despite available treatments. Understanding why cancer causes pain and how to treat it more effectively remains a critical challenge. The present investigation examines the latest research from 2022 to 2025 to understand the biological processes underlying cancer pain and to evaluate current treatment options. We found that cancer pain results from complex interactions between tumor cells, nerves, and the immune system. Modern treatments now include not only traditional pain medications but also advanced procedures. Some of these include nerve stimulation, targeted radiation therapy, and digital tools that use artificial intelligence to monitor and to predict pain. These findings highlight a shift toward personalized pain management strategies tailored to each patient’s specific needs, though challenges in implementing these innovations equitably across all healthcare settings remain.

## 1. Introduction

### 1.1. Epidemiology and Clinical Burden of Cancer Pain

Cancer pain remains one of the most prevalent, burdensome, and clinically challenging symptoms experienced by patients with malignancy, affecting an estimated 55–95% of individuals at advanced stages of disease [1,2]. Contemporary epidemiological data indicate that approximately two-thirds of patients with metastatic cancer experience moderate to severe pain despite advances in systemic therapies and supportive care, underscoring its persistent global impact on quality of life, functional capacity, and psychological well-being [3]. The burden extends far beyond individual suffering. Uncontrolled cancer pain is associated with reduced longevity, increased healthcare utilization, reduced adherence to anticancer treatments, and substantial socioeconomic costs [4].

### 1.2. Pathophysiological Mechanisms

From a mechanistic standpoint, cancer pain is now widely recognized as a prototypical form of mixed pain, where nociceptive, neuropathic, and, in a subset of patients, nociplastic mechanisms coexist [5]. The recognition of this mechanistic heterogeneity has emerged from recent advances in neurobiology, oncology, and immunology [6]. Tumor–neuron interactions have gained prominence, with studies demonstrating that malignant cells actively recruit and remodel neural pathways through neurotrophic factors, axonogenesis, and synaptic-like communication, thereby amplifying nociceptive transmission [7,8,9]. Parallel discoveries in neuroimmune crosstalk reveal that cytokines, chemokines, and glial activation contribute to sustained peripheral and central sensitization, shaping the transition from acute to chronic cancer pain [10,11]. In bone metastases, osteoclast activation releases acidic products and inflammatory mediators that sensitize acid-sensing ion channels (ASICs) and transient receptor potential vanilloid 1 (TRPV1) receptors on nociceptors, creating a unique pain phenotype characterized by movement-evoked breakthrough pain [12].

### 1.3. Current Management Approaches

Management strategies continue to evolve. International guidelines and recent updates reaffirm the WHO analgesic ladder, with paracetamol and non-steroidal anti-inflammatory drugs (NSAIDs) as the foundation for mild pain and analgesics such as anticonvulsants and antidepressants at all levels, used alone or in combination with opioids when appropriate [12]. Despite limited high-quality randomized controlled trial (RCT) evidence, NSAIDs remain widely used, particularly for bone and inflammatory components of cancer pain, often in combination with opioids, with careful consideration of gastrointestinal, renal, and cardiovascular risks and gastroprotection when indicated [13]. Opioids remain the cornerstone for moderate–severe cancer pain, endorsed by major societies such as the American Society of Clinical Oncology (ASCO) [14]. However, their use is increasingly framed within precision prescribing and opioid-sparing multimodal strategies, integrating adjuvant analgesics, interventional procedures, and supportive measures.

### 1.4. Digital Health and Remote Monitoring

At the same time, digital health and telemedicine solutions, ranging from telemonitoring systems to structured pain self-management apps, are emerging as tools to improve assessment, adherence, and timely titration of analgesic regimens [15]. However, implementation challenges include the digital divide (limited access for elderly or socioeconomically disadvantaged patients), incomplete validation of AI algorithms across diverse populations, data privacy concerns, and lack of reimbursement models.

### 1.5. Rationale and Scope of This Review

In this context of rapidly expanding mechanistic insight and therapeutic innovation, there is a clear need to synthesize recent advances in the pathophysiology and management of cancer pain, with particular emphasis on mixed-pain mechanisms, rational use of analgesics (RUA) (NSAIDs, anticonvulsants, antidepressants and opioids), adjuvant and interventional approaches, and the integration of digital technologies into patient-centered care.

## 2. Materials and Methods

### 2.1. Study Design

This study was conducted as a scoping review to systematically map recent evidence on mechanisms and management strategies for cancer-related pain. The methodology followed the framework originally proposed by Arksey and O’Malley and further refined by Levac et al. [16] and adhered to updated Joanna Briggs Institute (JBI) methodological guidance for scoping reviews. Reporting follows the PRISMA Extension for Scoping Reviews (PRISMA-ScR) [17].

### 2.2. Research Question

The overarching research question was: “How have biological mechanisms, pharmacological strategies, interventional and neuromodulatory procedures, radiotherapy, and digital/AI-enabled tools been investigated and applied in the management of cancer-related pain between 1 January 2022 and 30 September 2025?”

### 2.3. Eligibility Criteria

Eligibility criteria were defined a priori using the Population–Concept–Context (PCC) framework recommended by JBI. The population of interest included adults (≥18 years) experiencing cancer-related pain of any etiology (e.g., tumor-related, treatment-related, or mixed) across oncology, survivorship, and palliative-care settings. The concept encompassed a broad range of domains, including biological and neuroimmune mechanisms of cancer pain; pharmacological management with non-opioid analgesics, opioids, and adjuvant medications; interventional and neuromodulatory techniques such as nerve blocks, intrathecal therapies, spinal cord stimulation (SCS), dorsal root ganglion stimulation (DRGS), and peripheral nerve stimulation (PNS); radiotherapy approaches for pain palliation (e.g., cEBRT, SBRT, stereotactic modalities); and digital health solutions including remote monitoring, mobile health, wearables, and AI/ML or NLP applications for cancer-pain assessment, prediction, or management. The context included any healthcare setting, hospital, outpatient, home-based, or hospice, across all countries. Eligible evidence sources comprised primary quantitative, qualitative, or mixed-methods studies; secondary evidence such as systematic or scoping reviews; clinical practice guidelines; and high-quality observational cohorts or registries. Narrative commentaries and editorials were included only when they offered meaningful conceptual or mechanistic insights.

The review included articles published between 1 January 2020 and 30 September 2025, limited to the English language. Exclusion criteria were case reports or case series with fewer than five patients, conference abstracts without full text, non-human preclinical studies unless directly relevant to clinically applicable cancer-pain mechanisms, and articles in which pain was not a primary or explicitly analyzed outcome. Four electronic databases were analyzed: PubMed/MEDLINE, Embase, Scopus, and Web of Science Core Collection, supplemented by manual citation tracking of key reviews and guidelines. The final search was run on 20 November 2025.

### 2.4. Search Strategy

Search strategies were developed iteratively with reference to JBI guidance and the PRISMA-ScR explanation and elaboration paper [17]. For PubMed, Medical Subject Headings (MeSH) were combined with free-text terms related to cancer, pain, and each of the domains. The PubMed strategy was: ((“Neoplasms”[MeSH] OR neoplasm*[tiab] OR cancer*[tiab] OR malignan*[tiab] OR tumor*[tiab] OR tumour*[tiab]) AND (“Pain”[MeSH] OR “Pain Management”[MeSH] OR pain[tiab] OR analgesia[tiab] OR “cancer pain”[tiab])) AND (mechanis*[tiab] OR neuroimmune[tiab] OR “nerve sprouting”[tiab] OR “tumor-nerve”[tiab] OR opioid*[tiab] OR NSAID*[tiab] OR ibuprofen[tiab] OR adjuvant*[tiab] OR gabapentin*[tiab] OR duloxetine[tiab] OR “nerve block*”[tiab] OR “intrathecal”[tiab] OR “spinal cord stimulation”[tiab] OR “dorsal root ganglion”[tiab] OR “peripheral nerve stimulation”[tiab] OR “Radiotherapy”[MeSH] OR radiotherap*[tiab] OR “stereotactic body radiotherapy”[tiab] OR SBRT[tiab] OR “palliative radiotherapy”[tiab] OR “Telemedicine”[MeSH] OR “Mobile Applications”[MeSH] OR telemedicine[tiab] OR telehealth[tiab] OR “mobile app*”[tiab] OR mHealth[tiab] OR “Artificial Intelligence”[MeSH] OR “machine learning”[tiab] OR “natural language processing”[tiab] OR “clinical decision support”[tiab]) AND (“1 January 2020” [Date—Publication]: “30 September 2025” [Date—Publication]). For Embase, Scopus, and Web of Science, the strategy was translated using database-specific subject headings (e.g., Emtree terms for Embase) and proximity operators (e.g., cancer* NEXT/3 pain*) while preserving the same core concepts (cancer, pain, mechanisms, pharmacology, interventions, radiotherapy, digital/AI). Search results from all databases were exported into a reference-management software and de-duplicated. Title and abstract screening were performed independently by two reviewers (YVT, PVP) against the eligibility criteria. Potentially relevant records were retrieved in full text and assessed independently by the same reviewers. Discrepancies at either stage were resolved through discussion or consultation with a third reviewer.

**Figure 1:**
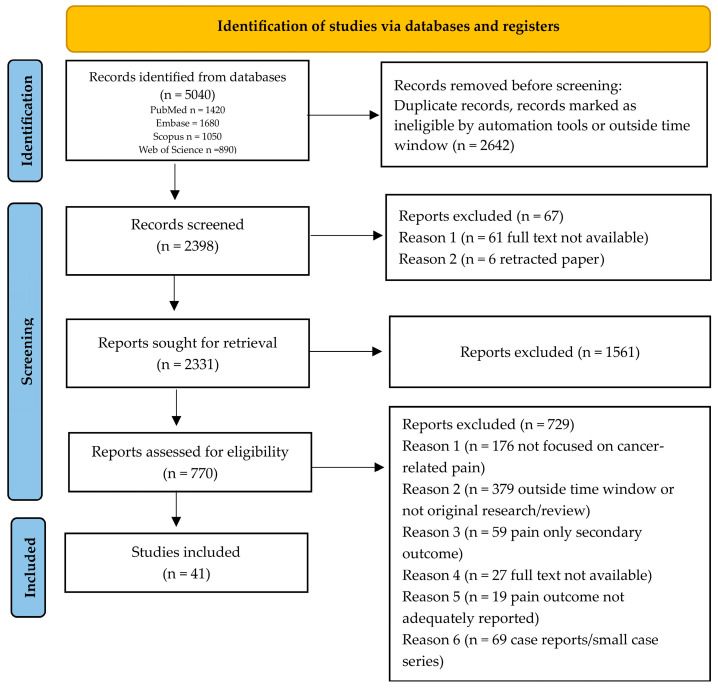
PRISMA flowchart.

The literature search identified a substantial body of contemporary evidence across cancer pain mechanisms and management. After screening for relevance and methodological rigor, the final selection comprised recent mechanistic studies, clinical trials, meta-analyses, interventional innovations, and technology-driven approaches suitable for qualitative synthesis. The comprehensive search across PubMed, Embase, Scopus, and Web of Science retrieved 5040 records. After removing 2642 duplicates or records marked ineligible by automation tools or outside the time window, 2398 records remained for title and abstract screening. Of these, 67 records were excluded due to full text not being available (n = 61) or being retracted (n = 6), leaving 2331 records for full-text retrieval. Following retrieval, 1561 records were excluded during screening. The full text of 770 articles was assessed for eligibility, of which 729 were excluded for reasons including lack of focus on cancer-related pain (n = 176), outside time window or not original research/review (n = 379), pain reported only as a secondary outcome (n = 59), full text not available (n = 27), inadequate pain outcome reporting (n = 19), or being case reports/small case series (n = 69). Ultimately, 41 studies were included in the scoping review (Figure 1).

## 3. Results

The qualitative analysis of the extracted literature revealed several coherent thematic groups reflecting the current evolution of cancer pain science. The first encompassed advances in biological mechanisms, including tumor–neuron interactions, neuroimmune activation, and sensitization processes. A second group comprised studies on pharmacological management, covering NSAIDs, opioids, antineuropathic agents, and emerging targeted analgesics. A third group focused on interventional and neuromodulatory approaches, detailing procedural innovations and device-based therapies. A fourth group captured radiotherapy developments relevant to painful metastases. Finally, an expanding body of work addressed digital health, remote monitoring, and AI-enabled tools, highlighting their growing integration into cancer pain assessment and personalized management.

### 3.1. Advances in Biological Mechanisms

Recent investigations have substantially advanced our understanding of the molecular and cellular processes underlying cancer-related pain, refining the concept of tumor–neuronal and neuroimmune crosstalk. First, the role of tumor–nerve interactions has become increasingly evident: malignant cells release neurotrophic factors such as nerve growth factor (NGF) and brain-derived neurotrophic factor (BDNF) and guidance cues (e.g., semaphorins, netrins) that stimulate axonal sprouting and afferent sensitization within the tumor microenvironment [18]. This nerve infiltration is directly implicated in pain genesis and correlates with disease aggressiveness [19,20].

Concurrently, neuroimmune signaling has emerged as a pivotal mechanism: activated nociceptors secrete neuropeptides like CGRP and substance P, which in turn modulate immune-cell function and perpetuate inflammatory signaling both peripherally and centrally [21]. For instance, recent data show that sensory neurons within the tumor milieu drive immune suppression and nociceptive sensitization through chemokine and cytokine networks [22]. The inflammation serves as the primary driving force underlying the transition from acute to chronic cancer pain through sequential inflammatory cascades at peripheral and central levels [23]. At the periphery, tumor-secreted algogens, including prostaglandins (PGE2), endothelin-1, glutamate, and protons, activate transient receptor potential (TRP) channels and acid-sensing ion channels (ASICs) on nociceptors, thereby amplifying afferent input and establishing peripheral sensitization [22]. This initial inflammatory phase is characterized by upregulation of pro-inflammatory cytokines including interleukin-1β (IL-1β), interleukin-6 (IL-6), and tumor necrosis factor-α (TNF-α) released by tumor-associated macrophages, neutrophils, and tumor cells themselves [23]. These cytokines directly modify nociceptor excitability by: increasing expression and membrane trafficking of voltage-gated sodium channels (Nav1.7, Nav1.8, Nav1.9); sensitizing TRPV1 and TRPA1 channels through protein kinase A and protein kinase C phosphorylation; and upregulating chemokine receptors (CCR2, CXCR4) that maintain nociceptor responsiveness to inflammatory mediators [24]. The transition to chronic pain becomes irreversible when peripheral neuroinflammation propagates centrally, triggering spinal neuroinflammation and central sensitization [25]. Persistent afferent barrage from peripherally sensitized nociceptors delivers sustained glutamate, substance P, and CGRP release onto dorsal horn neurons, initiating a cascade of glial cell activation [26]. Specifically, microglia undergo morphological transformation from ramified (surveillant) to amoeboid (activated) phenotype within 24–48 h of sustained nociceptive input [27]. Activated microglia release pro-inflammatory mediators including IL-1β, TNF-α, IL-6, chemokines (CCL2/MCP-1, CX3CL1/fractalkine), reactive oxygen species, nitric oxide, and brain-derived neurotrophic factor (BDNF) [21]. Microglial IL-1β activates neuronal IL-1 receptors, triggering intracellular signaling through MyD88-NFκB pathways that enhance NMDA receptor phosphorylation and membrane insertion, increasing synaptic strength [28]. Critically, microglial BDNF binds to neuronal TrkB receptors, activating downstream signaling that downregulates the potassium-chloride cotransporter KCC2, disrupting neuronal chloride homeostasis and converting GABA and glycine from inhibitory to excitatory neurotransmitters, a process termed “disinhibition” that fundamentally transforms spinal pain processing from balanced to hyperexcitable [29]. Astrocytes, activated secondary to microglial signals, amplify central sensitization through complementary mechanisms [30]. Astrocytic release of glutamate via hemichannels and ATP via pannexin channels provides additional excitatory drive to dorsal horn neurons [31]. Furthermore, astrocytes release thrombospondin-1 and D-serine, which promote excitatory synapse formation and potentiate NMDA receptor function, respectively [32]. The combined effect of microglial and astrocytic activation establishes a self-perpetuating neuroinflammatory state [33]. Importantly, once established, central sensitization becomes self-sustaining and largely independent of ongoing peripheral pathology—a phenomenon termed “pain centralization” or “maladaptive plasticity” [34]. This mechanistic understanding explains why cancer pain often persists or intensifies even when tumors respond to anticancer therapy and establishes neuroinflammation as a critical therapeutic target for preventing or reversing pain chronification. Recent evidence demonstrates that interventions targeting specific inflammatory mediators—including IL-1β antagonists, microglial inhibitors (minocycline, ibudilast), and inhibitors of chemokine signaling—can prevent or partially reverse established central sensitization in preclinical cancer pain models, though clinical translation remains limited [35].

Emerging research has identified substantial inter-individual variability in cancer pain susceptibility and analgesic response, partially attributable to genetic polymorphisms and epigenetic modifications [36]. Single nucleotide polymorphisms (SNPs) in genes encoding opioid receptors (OPRM1, OPRD1, OPRK1), catechol-O-methyltransferase (COMT), and voltage-gated sodium channels (SCN9A, SCN10A) have been associated with differential pain sensitivity and opioid dose requirements in cancer populations [37]. Beyond genetics, epigenetic alterations including DNA methylation, histone modifications, and microRNA dysregulation contribute to persistent pain states in cancer [38]. Hypermethylation of genes regulating endogenous opioid production or inhibitory neurotransmission may diminish descending pain modulation, while histone deacetylase activity influences transcription of pro-nociceptive mediators [39]. Specifically, DNA methylation patterns show critical regulatory roles: hypermethylation of the OPRM1 gene (encoding the μ-opioid receptor) has been associated with reduced receptor expression and diminished opioid analgesic efficacy in cancer patients [40,41]. Similarly, hypermethylation of the PENK gene (encoding proenkephalin, a precursor of endogenous opioid peptides) results in decreased endogenous opioid production, potentially compromising descending pain modulation [42,43]. Hypermethylation of GAD1 and GAD2 genes, which encode glutamic acid decarboxylase enzymes essential for GABA synthesis, may impair inhibitory neurotransmission in pain pathways, contributing to central sensitization [44,45]. Conversely, hypomethylation and subsequent overexpression of pro-nociceptive genes including SCN9A (Nav1.7 sodium channel), TRPV1 (capsaicin receptor), and IL6 (interleukin-6) have been documented in cancer pain states, amplifying nociceptive signaling [46]. Histone modifications further modulate pain-related gene transcription: histone deacetylase (HDAC) activity, particularly HDAC1 and HDAC2, suppresses acetylation of histone H3 and H4 at promoter regions of anti-nociceptive genes while permitting transcription of pro-nociceptive mediators such as TNF-α, IL-1β, and COX-2 [47]. Conversely, histone acetyltransferase (HAT) activity at promoters of genes encoding pronociceptive chemokines (CCL2, CXCL12) and inflammatory mediators contributes to sustained pain transmission [48].

MicroRNA dysregulation represents a third epigenetic mechanism in cancer pain. Upregulation of miR-21, miR-155, and miR-223 in tumor microenvironments and dorsal root ganglia promotes pro-inflammatory and pro-nociceptive signaling by targeting genes involved in pain resolution pathways [49]. For example, miR-21 targets PDCD4 (programmed cell death protein 4), enhancing NF-κB-mediated inflammatory signaling, while miR-155 suppresses SOCS1 (suppressor of cytokine signaling 1), potentiating cytokine-driven nociceptor sensitization [50]. Conversely, downregulation of miR-124 and miR-134 in cancer pain conditions results in disinhibition of their target genes including BDNF, CREB, and voltage-gated calcium channels (CACNA1C), thereby amplifying excitatory neurotransmission and synaptic plasticity in pain pathways [51]. Additionally, miR-200b downregulation permits increased expression of PKCε (protein kinase C epsilon), a critical mediator of nociceptor sensitization and hyperalgesia in cancer-induced bone pain [52].

The tumor microenvironment (TME) represents another complex ecosystem where malignant cells, immune infiltrates, stromal components, and neural elements engage in reciprocal signaling that amplifies pain [53]. Acidosis within the TME, resulting from dysregulated tumor metabolism and hypoxia, activates acid-sensing ion channels (ASICs) and transient receptor potential vanilloid 1 (TRPV1) on nociceptors, generating sustained afferent barrage [54]. Tumor-associated macrophages (TAMs) and myeloid-derived suppressor cells release not only classical inflammatory cytokines but also specialized pro-algesic lipid mediators including lysophosphatidic acid and sphingosine-1-phosphate [55]. Matrix metalloproteinases secreted by tumor and stromal cells liberate bioactive fragments from extracellular matrix proteins, further activating nociceptive pathways [24]. In bone metastases, osteoclast-mediated bone resorption releases protons, adenosine triphosphate and matrix-embedded growth factors that collectively activate periosteal and bone marrow nociceptors [56]. Nerve growth factor (NGF) is particularly abundant in metastatic bone lesions and binds to tropomyosin receptor kinase A (TrkA) on nociceptors, driving neuronal sensitization, sprouting, and phenotypic changes [57].

An emerging mechanism of tumor–neuron communication involves small extracellular vesicles (sEVs), or exosomes, lipid bilayer-enclosed nanovesicles secreted by cancer cells that transfer bioactive cargo-including proteins, mRNAs, microRNAs, and lipids to recipient cells [58]. Preclinical studies demonstrate that cancer-derived exosomes directly activate and sensitize nociceptors, contributing to cancer pain in a tumor-specific manner. In oral and head and neck cancer models, exosomes enriched with proteases and growth factors activate PAR2-dependent signaling, leading to TRPV1/TRPV4 sensitization, neuronal hyperexcitability, and mechanical allodynia; inhibition of exosome biogenesis or release markedly reduces pain behaviors, while exosome administration induces hypersensitivity in naïve animals, establishing their causal role [59,60,61]. In bone cancer pain, tumor-derived exosomes deliver autotaxin (ATX), promoting local production of lysophosphatidic acid (LPA), which activates LPA receptors on sensory neurons and drives nociceptive signaling; pharmacological inhibition of ATX attenuates mechanical hypersensitivity, validating the exosome–ATX–LPA axis as a therapeutic target [62]. Beyond direct nociceptor activation, cancer exosomes also promote axonogenesis, perineural invasion, and immune modulation within the tumor microenvironment, amplifying pain and disease progression [63].

Another emerging mechanism involves the growing evidence that the gut microbiota (GM) significantly contributes to the pathophysiology of cancer pain. Gut dysbiosis can disrupt mucosal barrier integrity, promote translocation of microbial pathogen-associated molecular patterns (PAMPs), and activate immune and glial pathways linked to neuroinflammation, thereby increasing pain sensitivity and contributing to both nociceptive and neuropathic pain phenotypes. Alterations in the GM have been associated with cancer cachexia, heightened pain sensitivity, reduced opioid efficacy, and the development of morphine-induced tolerance and hyperalgesia [64]. Specific microbial taxa and their metabolites, particularly short-chain fatty acids, have been shown to regulate systemic inflammatory responses and cytokine networks that influence neuro-immune communication. A recent translational review further highlighted the role of the microbiota, via the gut–brain axis and modulation of adaptive immunity, in shaping responses to cancer therapies and treatment-related adverse events [65]. Although research in this field is ongoing, current evidence suggests that the gut microbiota represents a novel modulator of tumor-associated neuroinflammation and a potential adjunctive therapeutic target in cancer pain management.

Collectively, these mechanistic insights recast cancer pain as a multifactorial disorder, rather than merely a consequence of tissue injury or nerve compression. Understanding this multi-layered interaction framework will be essential for translating mechanistic discoveries into more targeted and effective analgesic strategies [53].

### 3.2. Pharmacological Management

Pharmacological management of cancer-related pain must be grounded in mechanism-based analgesia and tailored to the mixed-pain profile typical of malignancy. Central to this strategy is the rational use of non-opioid analgesics, opioids, and adjuvant agents within a multimodal framework [12]. Non-opioid analgesics (e.g., paracetamol and NSAIDs) retain foundational status for mild to moderate pain and as adjuncts for more severe syndromes. The scoping review identified significant evolution in cancer pain pharmacotherapy beyond the traditional WHO analgesic ladder, including evidence challenging conventional approaches, emergence of targeted biological therapies, novel delivery systems, and mechanism-based precision medicine strategies. While opioids and non-opioid analgesics remain foundational, recent clinical trials and systematic evidence reveal both limitations of current practice and promising therapeutic innovations.

#### 3.2.1. Re-Evaluation of the WHO Analgesic Ladder

A pivotal 2021 multicenter RCT (n = 153) directly challenged the necessity of the WHO’s three-step ladder by comparing a two-step approach (paracetamol → strong opioid) versus the standard three-step approach (paracetamol → weak opioid → strong opioid) [66]. The trial demonstrated no significant difference in time to achieve stable pain control between groups (*p* = 0.42), with 53% of patients in the control group requiring escalation from step II to step III within two weeks due to inadequate analgesia. This finding challenges the clinical utility of weak opioids such as codeine and tramadol as an intermediate step in analgesic escalation, particularly in light of their unpredictable analgesic efficacy related to genetic polymorphisms in CYP2D6 that affect codeine bioactivation, the presence of ceiling effects that limit dose escalation, and their higher cost and reduced availability in many low- and middle-income countries when compared with low-dose morphine.

#### 3.2.2. NSAIDs Show Limited High-Quality Evidence but Retain Clinical Utility

Non-opioid analgesics (e.g., paracetamol and NSAIDs) retain foundational status for mild to moderate pain and as adjuncts for more severe syndromes. The ibuprofen exemplifies this class: although robust randomized controlled data in cancer pain remain scarce, clinical practice surveys indicate ibuprofen is the oral NSAID of choice in approximately 42.6% of UK palliative-care respondents [67]. Moreover, cancer-care algorithms list ibuprofen alongside other NSAIDs as first-line non-opioids in adult cancer pain management [68]. The theoretical basis for NSAIDs in cancer pain lies in modulation of cyclooxygenase (COX)-mediated prostaglandin synthesis and tumor-microenvironment inflammation, which may contribute to nociceptor activation and sensitization [69]. In prescribing ibuprofen or any NSAID in the cancer-pain setting, clinicians must carefully balance risk and benefit: gastrointestinal bleeding, renal impairment, platelet dysfunction, and chemotherapy-related thrombocytopenia are critical considerations. Guidelines emphasize caution in thrombocytopenic, renal-insufficient, or peri-operative oncology patients [70]. Evolving evidence suggests that NSAIDs might also impact oncologic outcomes (for example, improved survival associations with ibuprofen in observational cohorts) which raises the possibility of dual analgesic and disease-modifying benefit in selected settings [71]. However, high-quality analgesic trials of NSAIDs specifically in malignant pain remain absent; thus, they are best viewed as adjunctive rather than monotherapy in moderate to severe pain.

#### 3.2.3. Targeted Biological Therapies: Anti-NGF Monoclonal Antibodies

The most significant pharmacological advance identified in recent trials involves nerve growth factor (NGF) inhibition—a novel, non-opioid, mechanism-based approach targeting a key mediator of cancer-induced bone pain. NGF, released by tumor cells and tumor-associated macrophages in bone metastases, binds to tropomyosin receptor kinase A (TrkA) on nociceptors, driving peripheral sensitization, axonal sprouting, and phenotypic changes in sensory neurons [57]. A Phase III randomized, double-blind, placebo-controlled trial (NCT02609828, n = 155) evaluated tanezumab, a humanized monoclonal anti-NGF antibody, in patients with cancer pain predominantly from bone metastasis receiving background opioid therapy [72]. Patients received subcutaneous tanezumab 20 mg or placebo on Day 1 and Week 8. The trial met its primary endpoint at Week 8: least-squares mean change from baseline in daily average pain intensity at the index bone metastasis site was −2.03 with tanezumab versus −1.25 with placebo (difference −0.78, *p* = 0.0118) [NGF-4]. Secondary endpoints also favored tanezumab: 25.4% versus 12.3% achieved ≥30% pain improvement (*p* = 0.0405), and 22.5% versus 9.6% achieved ≥50% improvement (*p* = 0.0457) [NGF-4]. However, joint safety concerns emerged: 2 patients (2.8%) in the tanezumab arm developed pathologic fractures near pre-existing bone metastases, compared to none in placebo. Critically, development of all anti-NGF antibodies (tanezumab, fulranumab, fasinumab) was discontinued in 2021 following regulatory reviews concluding that joint safety risks outweighed benefits.

#### 3.2.4. Adjuvant Analgesics: Mechanism-Based Selection

The review identified growing evidence for mechanism-based adjuvant analgesic selection tailored to pain phenotype [73,74]:

Neuropathic cancer pain (nerve compression, chemotherapy-induced peripheral neuropathy): Gabapentinoids (gabapentin, pregabalin) demonstrated efficacy [75,76]. Serotonin–norepinephrine reuptake inhibitors (duloxetine and venlafaxine) have demonstrated clinical benefit in alleviating refractory neuropathic pain in patients with cancer [77].
Bone metastasis pain: Bisphosphonates (zoledronic acid, pamidronate) and RANKL inhibitors (denosumab) reduce osteoclast-mediated bone resorption, decreasing local acidosis and ATP release that activate periosteal nociceptors [78] demonstrate skeletal-related event reduction and modest analgesic benefit (number needed to treat 11 for pain reduction at 4 weeks) [79].
Corticosteroids: Dexamethasone reduces peritumoral edema and inflammatory mediator release (prostaglandins, cytokines), providing analgesic effects particularly for visceral pain, bone pain, and neuropathic pain from nerve compression. However, long-term use requires careful risk-benefit assessment given immunosuppression, hyperglycemia, and myopathy concerns in cancer populations [68].

#### 3.2.5. Opioid Optimization Strategies

Recent evidence highlights several complementary strategies aimed at optimizing opioid therapy in cancer pain while minimizing adverse effects and misuse. Pharmacogenetic-guided dosing further refines opioid selection and titration, as carriers of the OPRM1 A118G polymorphism typically require 20–30% higher doses to achieve comparable analgesia, while CYP2D6 genotype-guided strategies, such as avoiding codeine or tramadol in poor metabolizers and adjusting dosing in ultra-rapid metabolizers, have been shown to improve safety profiles [36]. In parallel, multimodal opioid-sparing regimens integrating adjuvant analgesics, regional anesthesia techniques, and interventional procedures reduce overall opioid requirements by 30–50% without compromising pain control, while also lowering the incidence of opioid-related adverse events [80].

An emerging non-opioid option for moderate-to-severe acute pain is suzetrigine, a first-in-class selective voltage-gated sodium channel NaV1.8 inhibitor approved by the U.S. Food and Drug Administration in January 2025 for the short-term management of acute pain [81]. Suzetrigine exerts its analgesic effects by selectively targeting peripheral nociceptive pathways, with minimal central nervous system penetration and no known addictive potential. In pivotal Phase 3 randomized trials in postoperative pain, suzetrigine demonstrated statistically significant reductions in pain intensity compared with placebo, with analgesic efficacy comparable to hydrocodone/acetaminophen and a favorable safety profile predominantly characterized by mild-to-moderate adverse events, including pruritus and muscle spasms [82]. Notably, regulatory approval was based exclusively on its efficacy in two well-established acute pain models: abdominoplasty, representing soft-tissue pain, and bunionectomy, representing bone-related pain, evaluated over a 48 h treatment period. Importantly, no clinical studies to date have assessed suzetrigine in chronic cancer-related pain and its current approval remains limited to short-term use in acute pain settings. Nevertheless, given its peripheral mechanism of action and opioid-sparing profile, suzetrigine represents a promising candidate for perioperative pain management in oncologic surgery, particularly in patients at increased risk of opioid-related adverse effects or dependence. Future studies are needed to explore its potential role in cancer-specific perioperative pain management strategies.

#### 3.2.6. Cannabinoids

Cannabinoids and cannabis-based medicines represent a potential adjunctive option for cancer pain management in patients with inadequate analgesia despite optimized opioid therapy. Through modulation of the endocannabinoid system, including CB1 and CB2 receptors, endogenous ligands, and metabolic enzymes, cannabinoids influence nociceptive transmission, neuronal excitability, and inflammatory signaling [83]. The most extensively studied preparation is nabiximols (THC:CBD oromucosal spray), which has demonstrated modest but statistically significant analgesic benefit in opioid-refractory cancer pain in randomized controlled trials, along with reduced use of breakthrough pain medication [84,85]. Meta-analytic evidence supports a small analgesic effect, though clinical impact is limited by dose-dependent adverse events and substantial heterogeneity across studies [86,87]. Observational data suggest potential reductions in pain severity and opioid consumption with medical cannabis use, but these findings are constrained by non-randomized designs [88]. Major evidence gaps remain, including a lack of high-quality, adequately powered randomized trials with standardized formulations and outcome measures, as highlighted by recent Cochrane evaluations [89]. Adverse effects, including cognitive impairment, dizziness, and sedation, are common; therefore, cannabinoids are not recommended as first-line therapy for cancer pain and should be reserved for carefully selected patients with refractory symptoms under specialist supervision [90,91]. Even though cannabinoids appear to be promising candidates for the treatment of cancer pain, future research priorities include the development of standardized cannabinoid formulations, optimization of THC:CBD ratios, evaluation of cannabinoid–opioid synergy, and identification of pharmacogenetic predictors of treatment response [89,92].

### 3.3. Neuromodulatory Approaches

In recent years, the management of cancer-related pain has increasingly incorporated interventional and neuromodulatory therapies, thereby extending beyond strictly pharmacological approaches. According to a 2023 scoping review, procedures such as intrathecal drug delivery systems (IDDS), percutaneous neurolytic blocks, vertebral augmentation (e.g., kyphoplasty/vertebroplasty), and minimally invasive neurosurgical interventions (e.g., cordotomy or dorsal-root entry zone lesioning) have gained prominence in oncologic pain settings [93]. These modalities offer both rapid analgesia and a potential opioid-sparing effect in patients with refractory pain or anatomical pain drivers [94]. Neuromodulation, specifically spinal cord stimulation (SCS), dorsal root ganglion stimulation (DRGS), and peripheral nerve stimulation (PNS), has emerged as a viable option for selected patients experiencing focal neuropathic or mixed pain syndromes in the context of malignancy [95,96]. For example, recent work indicates meaningful pain relief and improved functional outcomes in cancer-pain cohorts treated with SCS/DRGS [97]. In fact, this 2024 scoping review synthesizing 24 studies (16 on spinal cord stimulation, 7 on peripheral nerve stimulation, and 2 on dorsal root ganglion stimulation) demonstrated that spinal cord stimulation achieved substantial pain reduction, with mean Numeric Rating Scale (NRS) scores decreasing from 8.0 at baseline to 2.2 at a mean follow-up of 8.4 months, corresponding to a 72.5% reduction in pain intensity [97]. Cancer types demonstrating benefit from SCS/DRGS in recent series include lung, colorectal, breast, ovarian, mesothelioma, and renal malignancies, with pain etiologies encompassing metastatic bone pain, post-thoracotomy pain syndrome, and chemotherapy-induced peripheral neuropathy [98,99]. Meaningful pain relief was accompanied by improved functional outcomes: SCS produced significant opioid-sparing effects with average morphine equivalent daily dose (MEDD) decreasing from 1152.2 mg to 739.7 mg over 5 months (35.8% reduction), while a Memorial Sloan Kettering case series (n = 8) documented that 75% of patients were discharged within 2 days post-implantation and median opioid reduction reached 96 mg MEDD at 63-day follow-up [97]. DRGS, though less extensively studied, showed even greater pain reduction (NRS from 6.0 to 1.0, representing 83.3% reduction) over mean 6-month follow-up, particularly as salvage therapy after failed SCS [100].

In contrast, neuroablative neurosurgery remains a niche yet important component when pain is overwhelmingly refractory and life expectancy is limited. Percutaneous cervical cordotomy, which interrupts the lateral spinothalamic tract at C1–C2, has been evaluated in the largest national prospective UK data repository (n = 159 patients, 2012–2017), demonstrating that 67% of patients achieved ≥30% pain reduction with median worst pain decreasing from 9.0 to 5.0 (44% reduction) at median 9-day follow-up [101]. The most common indication was mesothelioma (57%), followed by lung cancer (18%), with median survival post-procedure of 1.3 months reflecting late referral in disease trajectory. Level 1 evidence from a randomized controlled trial (n = 16) confirmed that 85.7% (6/7) of cordotomy patients achieved >33% pain reduction at 1 week (median pain intensity decreased from 7 to 1, *p* = 0.022) compared to 0% in the continued palliative care control group, with 77.8% of control patients electing crossover to cordotomy [102]. A Brazilian 11-year series (n = 63) documented even more dramatic outcomes with mean NRS reduction from 8.4 to 0.78 (91% reduction), with particularly favorable results for lower limb and abdominal pain [103]. Serious adverse events remain rare (<3%), including temporary paresis, ataxia, respiratory dysfunction, and urinary retention [104]. Stereotactic anterior cingulotomy, which modulates the affective-emotional dimension of pain rather than pain intensity, has been systematically reviewed across 8 unique studies demonstrating that 32–83% of patients achieved meaningful pain relief, with optimal candidates being those with diffuse pain syndromes, head and neck malignancies, and significant emotional distress [105]. Recent case series documented dramatic opioid dose reductions: a 2023 series (n = 6) reported average daily oral morphine equivalent decreasing from 4411 mg pre-procedure to 240 mg at discharge (89% reduction) with 43% pain score reduction and no major complications [106], while a 2018 Israeli prospective series (n = 13) using double anterior cingulotomy reported 100% of patients experiencing substantial immediate post-operative pain relief [107]. Common transient adverse effects include urinary incontinence and confusion that resolve within days, while decline in focused attention may occur in the early post-operative period; however, long-term neurocognitive outcomes are generally favorable with median pain relief duration of 3–12 months [108]. Dorsal root entry zone (DREZ) lesioning, which ablates the dorsal horn entry zone to suppress hyperactive neurons, has been systematically evaluated in 46 studies (n = 1242 patients) with success rates varying by etiology: brachial plexus avulsion (60.8%), spinal cord injury (55.8%), phantom limb pain (35.3%), post-herpetic neuralgia (28.2%), and intractable cancer/post-radiation pain (effective but unpredictable outcomes) [109]. For cancer pain specifically, cervical DREZ has shown efficacy for post-radiation plexopathy and tumor infiltration of nerve roots, though outcomes are far more unpredictable than for brachial plexus avulsion and the procedure is not superior to intrathecal morphine for most cancer pain syndromes [110,111]. Mean complication rate across DREZ studies was 23.58%, including ataxia, paresis, bladder dysfunction, and rarely respiratory failure [112].

Selection criteria are stringent: interventions are reserved for individuals with confirmed pain pathway targets, minimal survival prognosis, and absence of alternative options, given their irreversible nature and risk-to-benefit ratio. Together, interventional and neuromodulatory techniques represent a mechanistically informed extension of cancer-pain management, targeting anatomical, neurophysiological, and neuropathic drivers of pain. Critical factors for success include precise pain phenotype identification, interdisciplinary collaboration (oncology, pain, neurosurgery), procedural timing relative to disease course, and coordination with systemic and adjuvant therapies. As the evidence base grows, these modalities are increasingly viewed not as last-resort options, but as integral components of personalized, advanced analgesic care in oncology [113].

### 3.4. Intrathecal Drug Delivery Systems

Intrathecal drug delivery systems (IDDS) are another well-established interventional option for refractory cancer pain, particularly in patients requiring high systemic opioid doses or experiencing intolerable opioid-related adverse effects [114]. By delivering preservative-free analgesics directly into the cerebrospinal fluid, IDDS bypass the blood–brain barrier and achieve high drug concentrations at spinal nociceptive receptors using substantially lower doses than systemic administration, thereby minimizing systemic opioid-related adverse effects and optimizing pain control in selected patients [115].

Multiple lines of evidence support IDDS efficacy in refractory cancer pain. A landmark randomized controlled trial by Smith et al. [116] demonstrated that IDDS provided superior pain control, reduced drug toxicity, and improved survival compared to comprehensive medical management in patients with refractory cancer pain (clinical success rate 84.5% vs. 70.8%, *p* = 0.06; drug toxicity 23% vs. 46%, *p* = 0.004)). Contemporary systematic reviews and health technology assessments confirm that IDDS achieves pain reduction in 60–80% of patients with moderate-to-low quality evidence (GRADE), with significant decreases in pain intensity maintained through 6- to 12-month follow-up [117]. A 2024 health technology assessment involving adults with cancer pain and life expectancy > 6 months demonstrated significant pain reduction compared to baseline (GRADE: Moderate to Low), alongside likely reductions in systemic opioid use (GRADE: Moderate to Low) and potential improvements in health-related quality of life, functional outcomes, and survival (GRADE: Low to Very Low) [118].

Additional real-world evidence is provided by a recent single-center retrospective study from a tertiary medical center in China, which evaluated IDDS use in patients with refractory cancer pain over a three-year period [119]. In this cohort of 96 patients, IDDS implantation resulted in a significant reduction in pain intensity, with mean NRS scores decreasing from 7.5 to 3.0 (*p* < 0.001), alongside high baseline opioid requirements (median oral morphine equivalent dose 290 mg/day). Despite advanced disease and limited life expectancy, median overall survival after implantation was 3 months, IDDS was associated with meaningful analgesic benefit and high family-reported satisfaction (75%). Real-world registry data from a prospective multicenter study (United States, Western Europe, Latin America) involving 1403 cancer pain patients showed significant improvements in pain scores at 6 months and 12 months compared to baseline, with EuroQol-5D quality of life scores demonstrating significant improvement at 6 months (*p* = 0.0016) [120]. Notably, 87% of patients whose registry follow-up ended were followed through death, with only 4.3% exiting due to device explant or therapy discontinuation, supporting therapy sustainability. The dramatic opioid-sparing effect of IDDS has been quantified in a large cohort study (n = 173) where pre-implant median daily oral morphine equivalent of 240 mg (range 0–2616 mg) decreased to a median of 0 mg 30 days postoperatively, with 72% of patients completely discontinuing systemic opioids and 85% reducing their oral morphine equivalent by ≥80% [121].

Appropriate patient selection is essential for the success of IDDS. The 2025 Polyanalgesic Consensus Conference (PACC^®^) guidelines, developed by the International Neuromodulation Society, provide evidence-based recommendations indicating that IDDS should be considered in cancer patients who experience inadequate analgesia despite appropriate escalation of systemic opioids and adjuvant therapies, or who develop intolerable opioid-related adverse effects that limit further dose escalation [122]. Candidates should have pain syndromes that are anatomically and mechanistically amenable to intrathecal therapy, typically focal or regional pain below the neck rather than diffuse metastatic disease, and with an anticipated prognosis sufficient to derive meaningful benefit from an invasive implant, balancing expected benefit against procedural risks. Importantly, contemporary practice emphasizes earlier IDDS implementation rather than “salvage therapy” after exhausting all systemic options, as earlier intervention may prevent opioid-induced hyperalgesia, preserve quality of life, and optimize functional status during remaining survival. Psychological evaluation is generally not required for cancer pain patients per PACC guidelines, though assessment of psychological and spiritual needs may facilitate comprehensive end-of-life care. Multidisciplinary team evaluation involving oncology, palliative care, pain medicine and neurosurgery optimizes candidate identification and therapeutic outcomes.

FDA-approved intrathecal agents for cancer pain include preservative-free morphine sulfate and ziconotide, a selective N-type calcium channel blocker. The PACC guidelines recommend medication selection based on pain phenotype and distribution. Morphine remains the first-line intrathecal therapy for cancer pain, typically initiated at low doses with titration according to prior opioid exposure and clinical response, while hydromorphone represents a viable alternative with greater potency. Ziconotide may be considered in patients with refractory neuropathic pain or in those at high risk for opioid-related adverse effects. In selected refractory cases, off-label agents such as fentanyl, sufentanyl, bupivacaine, or clonidine may be used, often in combination, although the quality of evidence declines with increasing polypharmacy [122]. Intrathecal trialing before permanent implantation remains controversial in cancer populations; while some centers perform routine screening trials (70.8% in one Chinese series) [119], the PACC panel considers trialing optional for cancer patients given disease progression uncertainty and urgency of pain control [122].

IDDS safety profiles are generally favorable, though complications require vigilant monitoring. Infection requiring surgical intervention (device explant, replacement, pocket revision, irrigation/debridement) occurs in approximately 3.2% of patients, representing the most common serious adverse event [123]. Device-related complications include catheter migration, disconnection, kinking, or fracture (5–10%), pump malfunction or battery depletion, and cerebrospinal fluid leakage. Pharmacological complications include pruritus, urinary retention, peripheral edema, and the rare but serious complication of intrathecal granuloma formation (inflammatory mass formation at catheter tip), which appears more common with higher morphine concentrations (>20 mg/mL) and prolonged therapy. Post-dural puncture headache occurs in <5% of patients and typically resolves within days. Device explantation rates remain low (4.3%), with most patients continuing therapy through death, reflecting acceptable risk-benefit profiles in this population [124].

However, despite demonstrated efficacy and safety, IDDS remains underutilized in cancer pain management due to barriers such as limited clinician awareness, restricted access to specialized centers, and regulatory or reimbursement challenges. Future efforts should focus on expanding access through multidisciplinary cancer pain programs, advancing intrathecal technologies and non-opioid agents, and evaluating earlier integration of IDDS to improve pain control while reducing opioid-related harms.

### 3.5. Radiotherapy Developments

Recent years have witnessed significant refinements in radiotherapy for cancer-related pain, particularly in the context of painful bone metastases [125]. Conventional external beam radiotherapy (cEBRT) continues to provide reliable palliation: a recent review reported pain-response rates of approximately 60–70% and complete relief in about 23–34% of treated cases [126]. Moreover, updated analyses show that both single- and multiple-fraction regimens yield equivalent overall response, although single-fraction treatments are associated with higher retreatment rates [127]. Advancing beyond conventional schedules, stereotactic body radiotherapy (SBRT) has gained increasing attention. A recent meta-analysis of eight randomized clinical trials (1090 patients) demonstrated that overall pain responses were comparable between SBRT and cEBRT; however, SBRT achieved significantly higher rates of complete pain relief at 1, 3 and 6 months (relative risk ranging from 1.4 to 2.5) [128]. These findings suggest that SBRT may confer superior durability of analgesia in selected patients, particularly those with oligometastatic disease or radioresistant histologies [127]. In parallel, there is growing emphasis on predictive modeling to personalize radiotherapy for pain relief. For example, a 2024 study developed a prognostic model integrating clinical parameters (primary tumor type, extent of metastases, steroid use) to stratify response likelihood in bone-metastasis patients undergoing palliative radiotherapy [129].

While radiotherapy primarily targets tumor and inflammatory mechanisms of skeletal pain, recent publications have also explored its mechanistic underpinnings: the analgesic effect may stem from modulation of periosteal nociceptor activity, reduction in tumor-induced osteolysis, suppression of inflammatory factors, and disruption of tumor–nerve crosstalk rather than mere mechanical stabilization [126]. Nonetheless, several open questions remain: optimal dose-fractionation schemes for complex or spinal lesions, timing of re-irradiation, integration with systemic analgesic regimens, and selection criteria for SBRT in routine pain-management algorithms. The recent update emphasizes the need for further high-quality trials to clarify dose, modality, and patient-selection variables [127].

Briefly, radiotherapy for cancer pain is evolving from standard palliation toward more precise, mechanism-based analgesic interventions. Its integration within multimodal pain management, including pharmacologic, interventional, and neuromodulatory strategies, positions it as a cornerstone of advanced cancer-pain care [130], even if with still some cautions [131].

### 3.6. Digital Health, Remote Monitoring, and AI-Enabled Tools

Digital health technologies have increasingly been integrated into cancer-pain management, reflecting a broader shift toward precision oncology and continuous symptom monitoring [132]. The COVID-19 pandemic accelerated the adoption of remote-care platforms, and subsequent evidence demonstrates that digital tools can enhance pain assessment, treatment adherence, and clinical decision-making for individuals living with cancer-related pain [133,134]. Recent reviews highlighted that digital interventions, including mobile applications, telemedicine platforms and wearable sensors, improve patient-reported pain outcomes, facilitate timely clinician intervention, and reduce unplanned healthcare use [135,136]. Mobile health (mHealth) systems have shown particular promise. For example, a controlled study evaluating a digital oncology pain-management platform reported significant reductions in pain intensity and distress, alongside improved self-management competence [137]. Remote monitoring platforms have also expanded the reach of palliative care. In a further review, telemedicine-based pain services demonstrated feasibility, high patient satisfaction, and comparable analgesic outcomes to in-person visits, particularly valuable for advanced cancer patients with limited mobility or in geographically underserved regions [138]. Wearable-sensor technologies represent an emerging frontier. A 2024 study demonstrated that continuous digital measurement of actigraphy-derived mobility indices correlates with fluctuations in cancer-pain severity, enabling dynamic monitoring and early detection of symptom escalation [139].

Artificial intelligence (AI) is increasingly incorporated into cancer-pain research and clinical management, particularly in the areas of pain prediction, phenotyping, and treatment optimization [135,140]. Machine-learning models have demonstrated the ability to integrate clinical, demographic, and patient-reported data to forecast clinically significant pain crises [141]. A 2023 study developed and validated a predictive model for severe cancer pain flares, achieving high discrimination accuracy and illustrating the potential of AI to support anticipatory, pre-emptive analgesic strategies [142]. Complementing these approaches, natural-language processing (NLP) methods have been applied to extract pain descriptors, severity scores, and temporal patterns from electronic health records [143]. This has been shown to improve the precision, completeness, and timeliness of pain assessment in oncology populations, offering scalable solutions for automated monitoring and clinical decision support [144]. Together, these developments signify a transition from episodic, clinic-based pain evaluation toward continuous, data-driven, personalized cancer-pain management. AI-enabled platforms enhance patient engagement, facilitate remote symptom monitoring, and open avenues for individualized prediction models that align analgesic interventions with dynamic patient needs [145].

Nevertheless, significant implementation challenges remain, including variation in digital literacy, interoperability and data-integration limitations, privacy and security concerns, and persistent disparities in access to digital health tools [146]. Addressing these barriers will be essential to ensure that AI-enabled cancer-pain management strategies are equitably integrated into routine oncology and palliative-care workflows, and to support future large-scale clinical validation.

**Figure 2:**
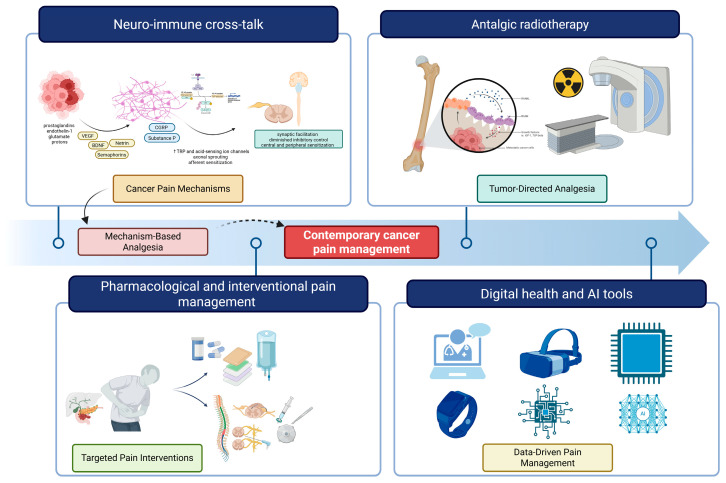
Integrated framework for contemporary cancer pain management. The figure illustrates four interconnected domains that constitute the modern approach to cancer-related pain: (1) Neuro-immune cross-talk, depicting the complex interactions between tumor cells, immune mediators, sensory neurons, and central sensitization pathways that drive pain pathogenesis; (2) Pharmacological and interventional pain management, encompassing non-opioid and opioid analgesics, adjuvant medications, nerve blocks, intrathecal delivery systems, and neuromodulation techniques; (3) Analgesic radiotherapy, showing targeted radiation delivery to painful bone metastases; and (4) Digital health and AI tools, representing emerging technologies including telemedicine platforms, wearable sensors, mobile applications, and artificial intelligence-enabled predictive models for continuous pain assessment and personalized treatment optimization. This integrated approach reflects the transition from empirical symptom management toward mechanism-based precision medicine in cancer pain care.

Figure 2 provides a schematic overview of the contemporary multimodal approach to cancer pain management, integrating mechanistic insights with therapeutic innovations across four key domains.

Table 1 summarizes the key clinical and translational studies included in this scoping review, spanning pharmacological, neuromodulatory, neuroablative, radiotherapeutic, and digital health-based approaches to cancer pain management while additional mechanistic and pathophysiological studies underpinning cancer pain, particularly those addressing tumor–neuron interactions, neuroimmune signaling, genetic and epigenetic modulation, exosome-mediated mechanisms, and treatment-related pain syndromes, are detailed in Supplementary Table S1.

## 4. Discussion

The examined recent evidence further strengthens the reconceptualization of cancer-related pain as a multifactorial, dynamically modulated syndrome, rather than a simple byproduct of tissue damage or nerve compression. An updated 2025 review proposes a multidimensional framework for cancer pain, integrating biological, pharmacologic, psychological, sociocultural, and functional domains, thereby reinforcing the need for personalized and mechanism-based treatment strategies [68].

Within this paradigm, advanced neurostimulation and neuromodulation techniques have gained traction. A recent scoping review focusing exclusively on cancer-induced pain reported that interventions such as SCS, DRGS and PNS can substantially reduce pain: average reductions in NRS pain scores described from 8.0 to 2.2 over follow-up, along with opioid-sparing effects in many cases [97]. Such data underscore the potential paradigm shift from opioids-centric analgesia to device-based, physiology-targeting pain control in oncology. Importantly though, the authors emphasize the low level of evidence (observational, retrospective), the heterogeneity of cases, and the lack of RCTs, highlighting the urgent need for prospective, controlled studies.

On the radiotherapy front, developments in precision techniques further refine the analgesic arsenal. In 2024, a series on stereotactic body radiotherapy (SBRT) for spinal metastases documented significant pain relief: more than half of patients achieved clinically meaningful pain reduction within 3 months post-treatment [147]. In separate work, robotic-guided spine SBRT was shown to be feasible, well tolerated, and associated with statistically significant improvements in pain and quality of life (*p* < 0.01) [148]. These findings suggest that SBRT, beyond its oncologic role, may serve as a durable and well-tolerated analgesic modality, especially in patients with oligometastatic or spinal bone disease.

While this review primarily focuses on pain arising directly from malignancy, cancer-related pain also includes a substantial treatment-related component that warrants explicit consideration. Among these syndromes, chemotherapy-induced peripheral neuropathy (CIPN) affects approximately 40% of patients receiving neurotoxic agents and represents a leading cause of persistent pain in cancer survivors, with duloxetine remaining the only FDA-approved pharmacological therapy with proven efficacy [149]. The global prevalence of chronic painful neuropathy and moderate-to-severe neuropathy among patients diagnosed with CIPN was estimated to be 47.76% [150]. CIPN pathophysiology is agent-specific and multifactorial, involving mitochondrial dysfunction, oxidative stress, neuroinflammation, and dysregulated chemokine signaling following exposure to platinum compounds, taxanes, vinca alkaloids, proteasome inhibitors, and immunomodulatory drugs [151]. Recent evidence has identified IRE1α stress-sensor activation as a mechanistic contributor to paclitaxel-induced neuropathy, highlighting potential predictive biomarkers and therapeutic targets [152]. In parallel, radiation-induced peripheral neuropathy (RIPN), although less frequent, is often progressive and irreversible, driven by microvascular injury, chronic inflammation, and radiation-induced fibrosis mediated by TGF-β1, ultimately resulting in axonal damage and ischemia [153,154]. Radiation-induced brachial plexopathy remains the most common manifestation, with incidence dramatically reduced by modern dose-fractionation strategies [154]. Post-surgical neuropathic pain syndromes, including post-mastectomy and post-thoracotomy pain, further contribute to the treatment-related pain burden [155]. Importantly, treatment-related pain syndromes differ fundamentally from tumor-related pain and require distinct management strategies, emphasizing neuropathic pain pharmacotherapy, neuromodulation, and preventive approaches rather than tumor-directed therapies alone [68]. As cancer survivorship continues to increase, treatment-related pain is expected to represent a growing proportion of the overall cancer pain burden, underscoring the need for integrated survivorship-focused pain management models [156].

Concurrently, the field of digital health and AI-enabled pain management in oncology is gaining momentum. A 2024 systematic review compiled 44 studies (2006–2023) investigating machine learning (ML) and AI applications for cancer-related pain: models showed promising performance in classifying pain after cancer therapy or during active disease (median AUC 0.80–0.86), and some attempted to support pain management decisions [136]. Additional work combined radiomics and NLP to successfully identify pain signatures on radiotherapy simulation CT scans, suggesting that unsupervised and multimodal AI pipelines may soon enable scalable, imaging-based pain assessment [157]. Yet, despite these advances, critical challenges remain. For neuromodulation, the lack of randomized trials, small sample sizes, and patient heterogeneity limit generalizability. For SBRT, optimal fractionation schemes, re-irradiation criteria, and integration with systemic therapy still lack robust evidence. And for AI-based tools, key obstacles include external validation, data privacy/interoperability, and digital-health literacy, which may limit equity in access. Indeed, the most recent comprehensive evaluation of AI/ML in cancer pain, while optimistic, notes that only 14% of models had external validation, and only 23% had direct clinical application [136].

Cancer pain represents a distinct and uniquely complex pain state that differs fundamentally from classical inflammatory and neuropathic pain, explaining the persistent limitations of current analgesic strategies. It is intrinsically mechanistically heterogeneous, combining nociceptive, neuropathic, and cancer-specific processes that evolve dynamically with disease progression, including tumor–nerve interactions, release of pro-algesic mediators, pathological bone remodeling, neuroinflammation, ischemia, and aberrant neuronal sprouting [23,155]. Clinically, cancer pain manifests as a triad of background, breakthrough, and incident pain, each with distinct therapeutic challenges and often limited responsiveness to standard analgesics [158]. Approximately 40% of cancer pain involves neuropathic mechanisms, yet cancer-related neuropathic pain engages distinct neuronal pathways and central neuroplastic changes that differ from non-cancer pain states, rendering conventional treatments suboptimal [159,160]. These biological complexities, together with patient heterogeneity, evolving disease trajectories, and methodological limitations of cancer pain trials, underscore the need for mechanism-based phenotyping and cancer-specific therapeutic strategies rather than extrapolation from non-cancer pain models [161,162].

Overall, evidence from 2022–2025 reinforces that cancer pain management is fundamentally a multidisciplinary, precision-medicine–oriented approach. However, a key priority remains the consistent implementation of this collaborative paradigm across diverse healthcare settings, including resource-limited hospitals and community practices, where substantial barriers persist. These include limited access to multidisciplinary pain teams, insufficient training in mechanism-based pain assessment, geographic disparities in the availability of interventional techniques, and fragmentation within healthcare systems. AI-enabled technologies and digital health platforms have the potential to strengthen and extend this established collaborative model by facilitating access to specialized expertise in underserved settings. Nevertheless, to translate this potential into routine oncology and palliative care practice, prospective controlled trials, standardization of interventions, and rigorous external validation of AI-based tools will be essential.

Limitations: This scoping review has several methodological limitations. First, consistent with scoping-review guidance, no formal risk-of-bias assessment was undertaken, limiting conclusions regarding the quality or comparative strength of evidence. Second, the broad scope spanning five domains introduces substantial heterogeneity in study designs, populations, and outcomes, precluding quantitative synthesis. Third, although four major databases were searched, relevant studies published in other repositories or non-English languages may have been missed. Fourth, the inclusion of diverse evidence types, including observational and technological studies, increases variability in reporting standards. Finally, the rapidly evolving fields of neuromodulation and AI may lead to evidence quickly becoming outdated.

## 5. Conclusions

This scoping review shows that cancer pain management is shifting from empirical symptom control toward mechanism-based, precision-oriented care. Contemporary evidence highlights cancer pain as a multifactorial syndrome driven by tumor–neuron–immune interactions and modulated by individual genetic and epigenetic susceptibility. Therapeutic strategies now encompass advanced neuromodulation, precision radiotherapy, interventional procedures, and digital health-enabled monitoring alongside foundational analgesics. Persistent gaps include limited high-quality trials, insufficient validation of AI-based tools, and inequitable access to novel therapies. Advancing the field requires rigorous prospective studies, methodological standardization, and implementation science to ensure that precision cancer pain management is accessible and clinically effective for all patients.

## Figures and Tables

**Table 1:** Key Clinical and Translational Studies Examined in the Research.

Domain	Study (Year)	Design/n	Intervention/Topic	Key Findings	Clinical Significance
Pharmacological interventions	WHO ladder re-evaluation	Multicenter RCT/153	Two-step vs. three-step WHO ladder	No difference in time to pain control; >50% required escalation to strong opioids	Challenges necessity of weak-opioid step
	Fallon et al., 2023 [72]	Phase III RCT/155	Tanezumab (anti-NGF)	Greater NRS reduction vs. placebo; fracture risk near metastases	Proof-of-concept; safety limits use
	Bertoch et al., 2025 [82]	Phase 3 RCT/2191	Suzetrigine (Nav1.8 inhibitor) for postoperative pain (abdominoplasty, bunionectomy)	Suzetrigine vs. placebo: 48.4 (abdominoplasty, *p* < 0.0001) and 29.3 (bunionectomy, *p* = 0.0002); comparable to hydrocodone/acetaminophen; AEs mild-moderate (pruritus, muscle spasms)	First FDA-approved selective Nav1.8 inhibitor for acute pain; non-opioid with peripheral mechanism, no addictive potential
Neuromodulation	Crowther et al., 2022 [98]	Retrospective cohort/28	Spinal cord stimulation (SCS)	NRS 8.0 → 2.2; opioid reduction	Viable option for refractory cancer pain
	Bulat et al., 2023 [99]	Case series/8	SCS	Rapid discharge; sustained opioid reduction	Feasible and safe in cancer patients
	Chung et al., 2025 [100]	Case series	DRGS vs. SCS	Greater pain reduction with DRGS	DRGS promising for selected syndromes
	Vu et al., 2025 [97]	Scoping review/24 studies	SCS, DRGS, PNS	Pain and functional improvement across cancers	Supports neuromodulation as multimodal care
Intrathecal drug delivery systems (IDDS)	Wang et al., 2024 [119]	Retrospective cohort/96	IDDS for refractory cancer pain at Chinese tertiary center	Mean NRS 7.5→3.0 (*p* < 0.001); median baseline OME 290 mg/day; 70.8% intrathecal trial; median survival 3 months; 75% family satisfaction	Real-world outcomes in advanced disease with high baseline opioid requirements
	Stearns et al., 2020 [120]	Prospective multicenter registry/1403	IDDS (US, Europe, Latin America)	Pain scores improved at 6 months and 12 months; EuroQol-5D improved; 87% followed through death; 4.3% explant; 3.2% infection	Largest real-world registry; sustained efficacy and high therapy retention through end of life
	Sindt et al., 2020 [121]	Retrospective cohort/173	IDDS opioid-sparing effects in advanced cancer	Pre-implant median OME 240 mg/day→0 mg at 30 days; 72% discontinued systemic opioids; 85% reduced OME ≥ 80%	Dramatic systemic opioid-sparing effect; IDDS as replacement for high-dose systemic opioids
	Deer et al., 2025 (PACC) [122]	Consensus guidelines/Expert panel	PACC 2025 updates on IDDS for cancer pain	Evidence-based recommendations for patient selection, drug algorithms, comorbidity management; earlier implementation vs. salvage therapy	International standard for IDDS best practices; algorithmic approach to medication selection
Neuroablative procedures	Doyle et al., 2022 [101]	RCT/16	Cordotomy vs. standard care	Superior pain relief vs. control	Level-1 evidence for cordotomy
	Adams et al., 2023 [105]	Systematic review	Cingulotomy	32–83% meaningful pain relief	Option for diffuse/affective pain
Radiotherapy	Bindels et al., 2024 [128]	Meta-analysis/1090	SBRT vs. cEBRT	Similar response; SBRT faster and more durable	Precision RT for bone metastases
	Rossi et al., 2024 (PRAIS) [129]	RCT secondary analysis	Palliative radiotherapy	Predictors of poor pain response identified	Enables risk stratification
	Chou et al., 2024 [147]	Retrospective cohort	Spine SBRT	Durable pain relief; favorable safety	Supports SBRT for spinal pain
Digital health & AI	Salama et al., 2024 [136]	Systematic review/44	AI/ML for cancer pain	Pain prediction AUC 0.75–0.92	Feasible; needs validation
	Bang et al., 2023 [142]	ML retrospective	Deep learning prediction	Accurate prediction of pain flares	Enables proactive pain management
	Hamdoune et al., 2024 [132]	Systematic review	Digital health tools	Reduced pain and distress	Supports telemedicine integration
Mechanistic/translational	Martel Matos et al., 2022 [22]	Translational/preclinical	sEVs in head & neck cancer	sEVs necessary and sufficient for pain	New therapeutic targets
	Fan et al., 2024 [18]	Preclinical study	Exosome–ATX–LPA axis	ATX inhibition reduces bone cancer pain	Druggable mechanism identified

## Data Availability

No new data were created or analyzed in this study.

## Supplementary Materials

The following supporting information can be downloaded at: https://www.mdpi.com/article/10.3390/cancers18020259/s1, Table S1. Mechanistic and pathophysiological evidence underlying cancer pain.

## Abbreviations

The following abbreviations are used in this manuscript:

AI	Artificial Intelligence
ASCO	American Society of Clinical Oncology
ASIC	Acid-Sensing Ion Channel
ATX	Autotaxin
BDNF	Brain-Derived Neurotrophic Factor
CB1	Cannabinoid Receptor 1
CB2	Cannabinoid Receptor 2
CBD	Cannabidiol
cEBRT	Conventional External Beam Radiotherapy
CGRP	Calcitonin Gene-Related Peptide
CIPN	Chemotherapy-Induced Peripheral Neuropathy
COMT	Catechol-O-Methyltransferase
COVID-19	Coronavirus Disease 2019
COX	Cyclooxygenase
DNA	Deoxyribonucleic acid
DREZ	Dorsal Root Entry Zone
DRGS	Dorsal Root Ganglion Stimulation
EBRT	External Beam Radiotherapy
FDA	Food and Drug Administration
GAD1	Glutamic Acid Decarboxylase 1
GAD2	Glutamic Acid Decarboxylase 2
HDAC	Histone Deacetylase
IDDS	Intrathecal Drug Delivery Systems
IL	Interleukin
JBI	Joanna Briggs Institute
LPA	Lysophosphatidic Acid
MDSC	Myeloid-Derived Suppressor Cell
MEDD	Morphine Equivalent Daily Dose
MeSH	Medical Subject Headings
mHealth	Mobile Health
miRNA	MicroRNA
ML	Machine Learning
mRNA	Messenger RNA
MSK	Memorial Sloan Kettering
NGF	Nerve Growth Factor
NLP	Natural Language Processing
NNH	Number Needed to Harm
NNT	Number Needed to Treat
NRS	Numeric Rating Scale
NSAID	Non-Steroidal Anti-Inflammatory Drug
OME	Oral Morphine Equivalent
OPRD1	Delta-Opioid Receptor Gene
OPRK1	Kappa-Opioid Receptor Gene
OPRM1	Mu-Opioid Receptor Gene
PAR2	Protease-Activated Receptor 2
PCC	Population–Concept–Context
PENK	Proenkephalin Gene
PNS	Peripheral Nerve Stimulation
PRAIS	Palliative Radiotherapy and Inflammation Study
PRISMA-ScR	Preferred Reporting Items for Systematic Reviews and Meta-Analyses Extension for Scoping Reviews
QOL	Quality of Life
RANKL	Receptor Activator of Nuclear Factor Kappa-B Ligand
RCT	Randomized Controlled Trial
RIPN	Radiation-Induced Peripheral Neuropathy
RNA	Ribonucleic Acid
RUA	Rational Use of Analgesics
SBRT	Stereotactic Body Radiotherapy
SCN9A	Voltage-Gated Sodium Channel 9A Gene
SCS	Spinal Cord Stimulation
sEVs	Small Extracellular Vesicles
SNP	Single Nucleotide Polymorphism
TAM	Tumor-Associated Macrophage
THC	Tetrahydrocannabinol
TME	Tumor Microenvironment
TNF	Tumor Necrosis Factor
TrkA	Tropomyosin Receptor Kinase A
TRP	Transient Receptor Potential
TRPV1	Transient Receptor Potential Vanilloid 1
TRPV4	Transient Receptor Potential Vanilloid 4
WHO	World Health Organization
