# Supplementary material for: Advances in the Pathophysiology and Management of Cancer Pain: A Scoping Review
Source: Cancers (Basel). 2026 Jan 14;18(2):259. doi: 10.3390/cancers18020259 (PMC12838970; doi:10.3390/cancers18020259)
Supplement: Supplementary file 1 [file cancers-18-00259-s001.zip › cancers-4048090-supplementary.pdf]

Supplementary Table S1. Mechanistic and pathophysiological evidence underlying cancer pain.

Domain	Study (Year)	Design	Model / Focus	Key Mechanisms Identified	Relevance to Cancer Pain
**Tumor–neuron interactions**	Fan et al., 2024 [18]	Narrative review	Solid tumors	NGF/BDNF release, axonal sprouting, nociceptor sensitization	Establishes bidirectional tumor–nerve signaling
	Yang et al., 2022 [10]	Narrative review	Multiple cancers	Neural remodeling and neurotrophic signaling	Explains tumor-driven pain amplification
**Perineural invasion**	Yoneda et al., 2023 [19]	Review	Bone and solid tumors	Nerve invasion correlates with pain severity and poor prognosis	Common mechanism across cancer types
	Shaikh et al., 2025 [20]	Review	Pancreatic and head/neck cancer	Perineural invasion drives neuropathic pain	Explains refractory pain phenotypes
**Neuroimmune mechanisms**	Ma et al., 2023 [7]	Review	Tumor microenvironment	Macrophage–neuron crosstalk, cytokine release	Inflammatory–neuropathic overlap
	Mardelle et al., 2024 [8]	Review	CNS/PNS	Microglial and astrocytic activation	Central sensitization
	Haroun et al., 2022 [23]	Review	Cancer pain models	Mast cell and T-cell signaling	Immune-driven nociception
**Central sensitization**	Varrassi et al., 2025 [25]	Review	Spinal cord	Astrocyte activation, BDNF–TrkB signaling	Sustained pain despite treatment
	Xiong et al., 2024 [11]	Review	Preclinical models	Altered dorsal horn excitability	Explains pain chronification
	Wang et al., 2025 [34]	Review	Cancer pain models	Synaptic plasticity changes	Distinct from non-cancer pain
**Genetic & epigenetic modulation**	Bugada et al., 2020 [36]	Review	Clinical cohorts	OPRM1, COMT polymorphisms	Interindividual analgesic response
	Ho et al., 2020 [37]	Review	Epigenetics	DNA methylation, HDAC activity	Pain persistence
	Rembiałkowska et al., 2025 [38]	Review	microRNAs	miR-21, miR-124 regulation	Neuroinflammatory modulation
**Exosome-mediated signaling**	Dubeykovskaya et al., 2022 [60]	Translational	Oral cancer	Tumor exosomes activate PAR2→TRPV1	Novel tumor–neuron pathway
	Cata et al., 2022 [58]	Translational / preclinical	Head & neck cancer	sEVs necessary and sufficient for pain	New therapeutic targets
	Khasabova et al., 2023 [62]	Preclinical	Bone cancer	Exosome–ATX–LPA signaling	Druggable pain mechanism
**Treatment-related pain syndromes**	Desforges et al., 2023 [149]	Review	CIPN	~40% prevalence; neuroinflammation	Persistent survivorship pain
	Delanian et al., 2012 [154]	Review	RIPN	Fibrosis-driven neuropathy	Often irreversible pain
